# Supplementary material for: Placental multi-omics integration identifies candidate functional genes for birthweight
Source: Nat Commun. 2022 May 2;13:2384. doi: 10.1038/s41467-022-30007-1 (PMC9061712; doi:10.1038/s41467-022-30007-1)
Supplement: Supplementary file 1 — Supplementary Information [file 41467_2022_30007_MOESM1_ESM.pdf]

SUPPLEMENTARY INFORMATION

Placental multi-omics integration identifies candidate functional genes for birthweight

Fasil Tekola-Ayele*, Xuehuo Zeng, Suvo Chatterjee, Marion Ouidir, Corina Lesseur, Ke Hao, Jia Chen, Markos Tesfaye, Carmen J. Marsit, Tsegaselassie Workalemahu, Ronald Wapner

* Corresponding author: Fasil Tekola-Ayele (ayeleft@mail.nih.gov), Division of Population Health Research, Division of Intramural Research, *Eunice Kennedy Shriver* National Institute of Child Health and Human Development, National Institutes of Health, Bethesda, MD, USA

SUPPLEMENTARY FIGURES

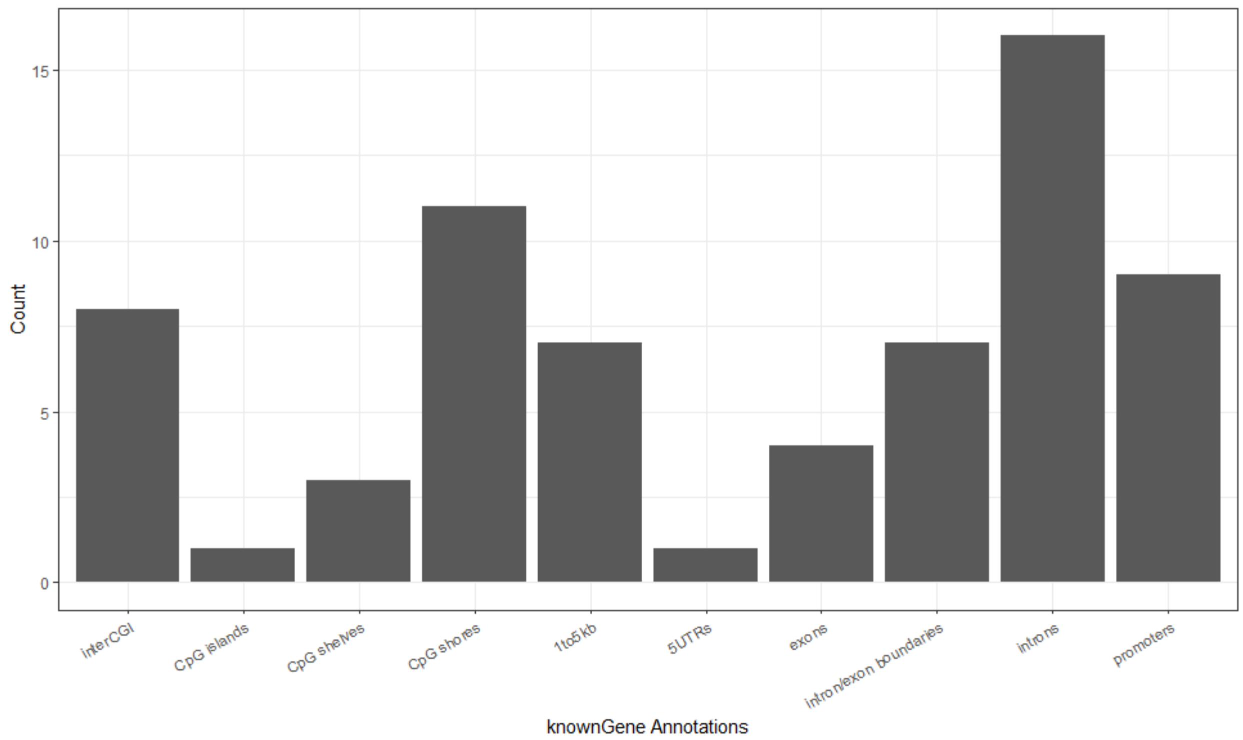

Figure S1. Number of DNA methylation CpG sites per regulatory annotation. The DNA methylation sites found to be colocalized with gene expression and birthweight GWAS loci (presented in Table 1) were annotated using the *annotatr* R package, Cavalcante RG, Sartor MA (2017). annotatr: genomic regions in context. Bioinformatics. R package version 1.20.0.

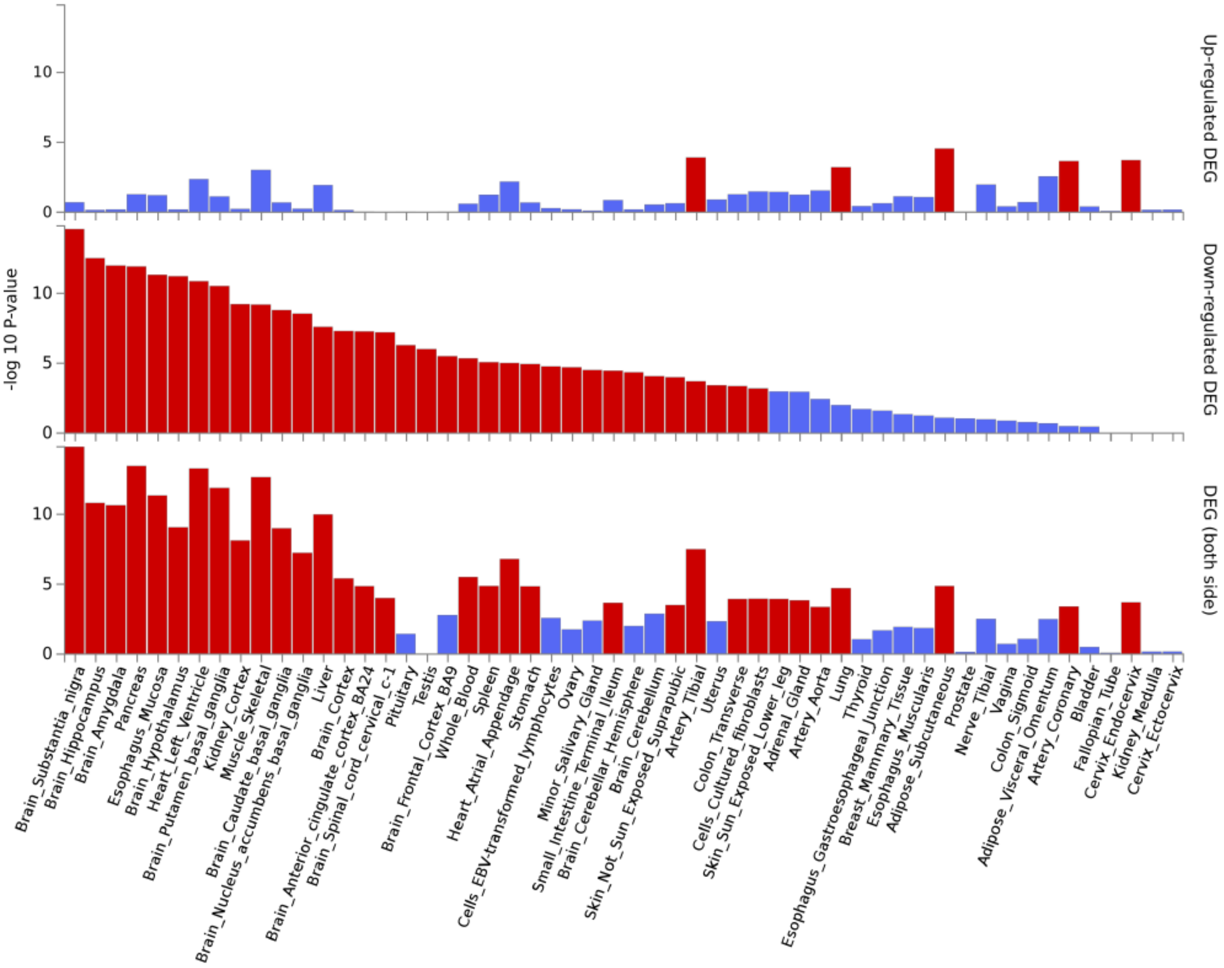

Figure S2. Enrichment of 402 genes annotating mQTL SNPs and their target DNAm sites in placenta. Significantly enriched DEG sets ($P_{bon} < 0.05$) are highlighted in red. The gene list was significantly enriched for downregulated differential gene expression in 34 GTEx tissues (Bonferroni-adjusted P <0.05) and for upregulated gene expression in 5 GTEx tissues. The strongest enrichment for downregulation was detected in brain and the strongest enrichment for upregulation was observed in subcutaneous adipose tissue.

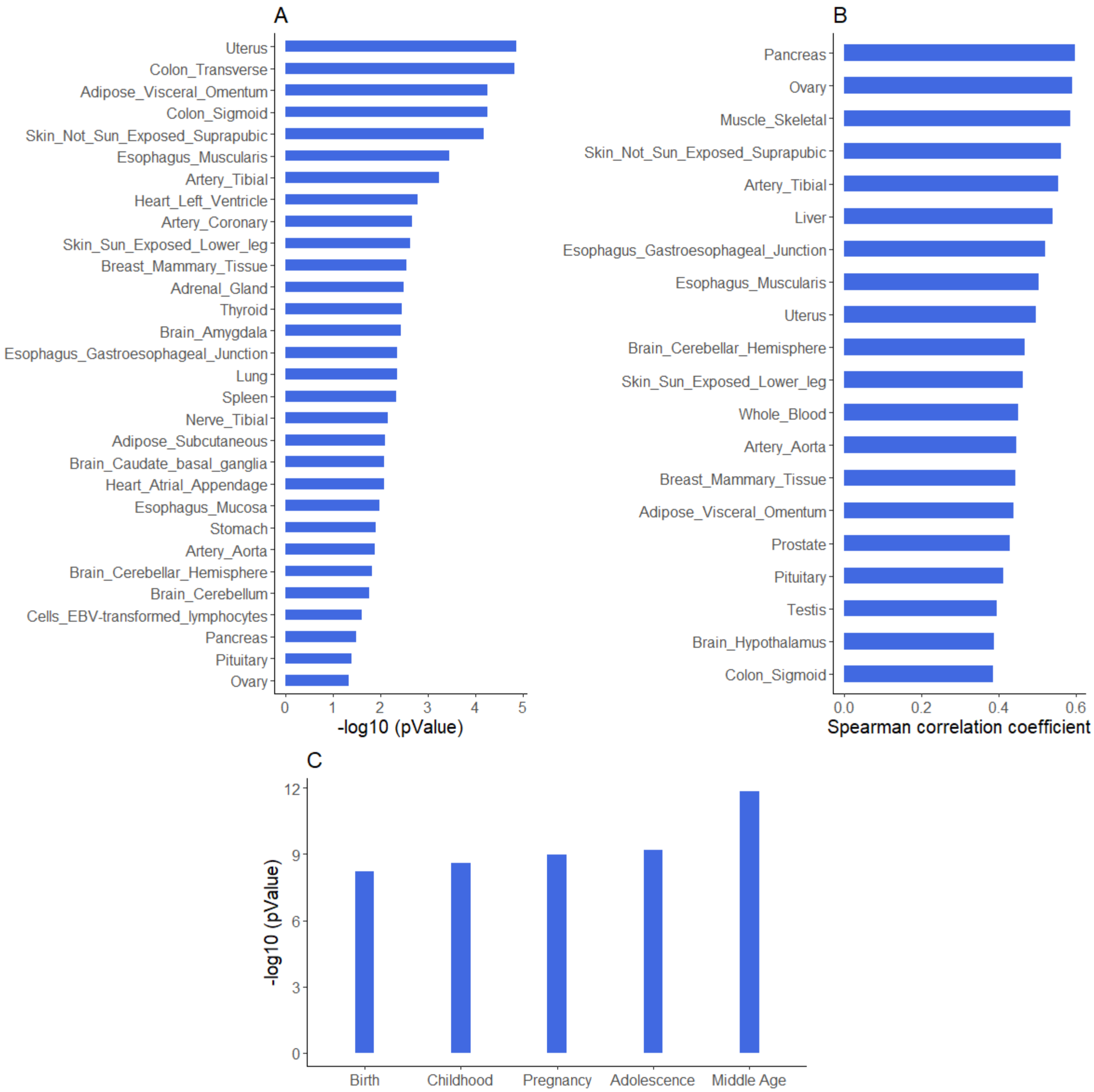

Figure S3. Enrichment of *cis*-eQTL and *cis*-mQTL in non-placental tissues for birthweight-associated genetic variants with significant QTL evidence in placenta. Birthweight GWAS loci that were also eQTL in placenta (n=26 SNPs), compared to birthweight GWAS loci that were not eQTL in placenta (n=247 SNPs), were significantly more likely to be eQTL in 30 GTEx tissues shown in (**A**) in descending magnitude of statistical significance based on a one-sided hypergeometric test nominal *P*-value. Their eQTL association coefficients in placenta were correlated (nominal *P*-value <0.05, two-sided Spearman correlation test) with their eQTL association coefficients in 20 GTEx tissues shown in **(B)**, the strongest correlations being with pancreas, ovary, and skeletal muscle and the weakest correlations being with several brain tissue types. Birthweight GWAS loci that were also mQTL in placenta (n=158 SNPs), compared to birthweight GWAS loci that were not eQTL in placenta (n=115 SNPs), were more likely to be mQTL in blood samples at birth, childhood, adolescence, middle-age and pregnancy from the ARIES mQTL database based on a one-sided test nominal *P*-value <0.05 (**C**), with the evidence for association strengthening as age increases across the lifespan.

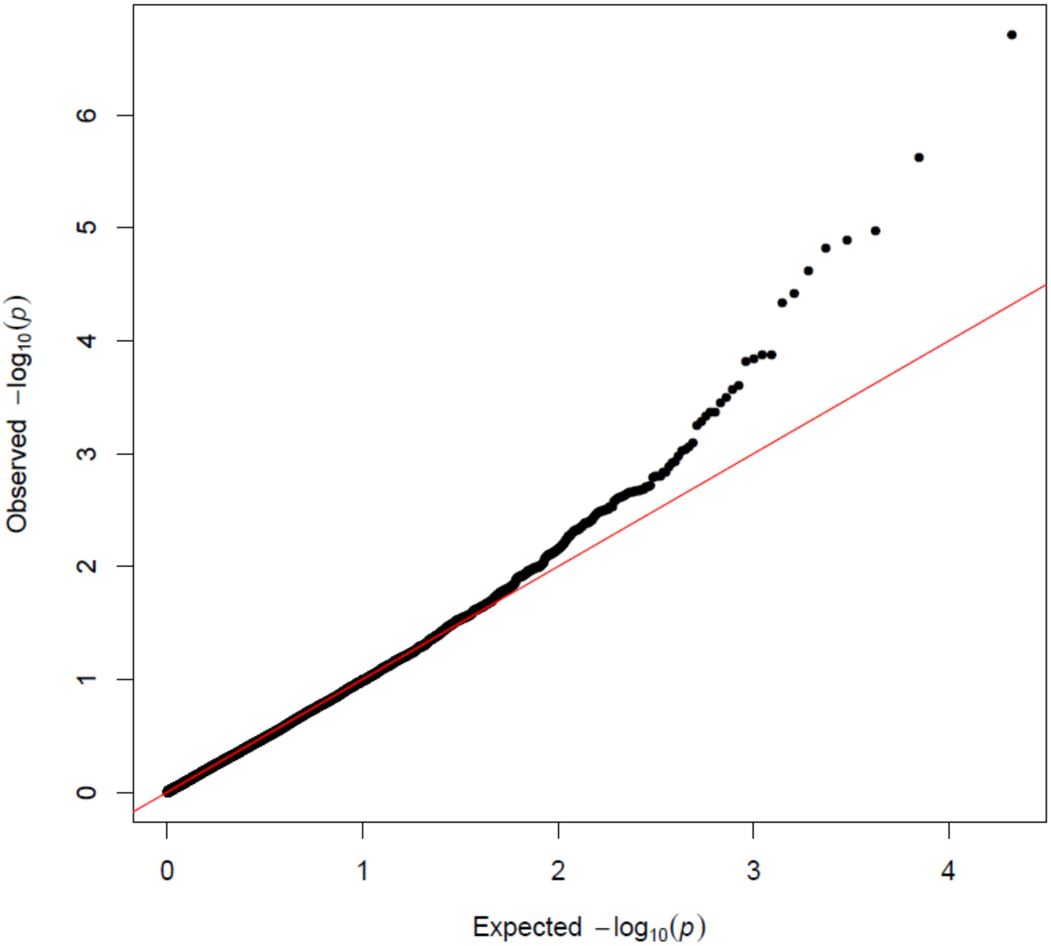

Figure S4. Quantile-Quantile (QQ) plot of p-values resulting from *cis*-eQTL mapping of the 273 birthweight-associated SNPs and 7901 genes within 1MB distance from the SNPs. With λ=0.926, the plot showed absence of inflation.

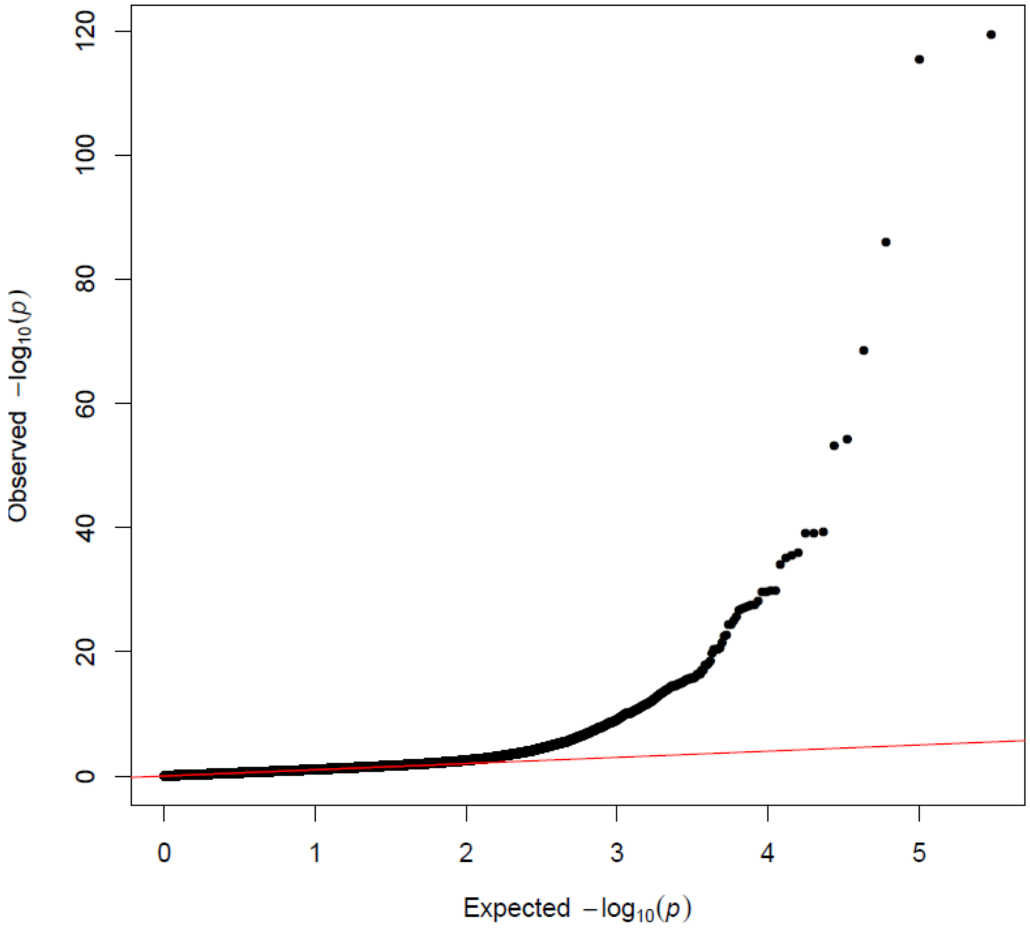

Figure S5. Quantile-Quantile (QQ) plot of p-values resulting from *cis*-mQTL mapping of the 273 birthweight-associated SNPs and 104,053 CpG DNA methylation (DNAm) sites found within 1 Mb distance from the SNPs. With λ=0.993, the plot showed absence of inflation.
