# Supplementary material for: Placental multi-omics integration identifies candidate functional genes for birthweight
Source: Nat Commun. 2022 May 2;13:2384. doi: 10.1038/s41467-022-30007-1 (PMC9061712; doi:10.1038/s41467-022-30007-1)
Supplement: Supplementary file 3 — Description of Additional Supplementary Files [file 41467_2022_30007_MOESM3_ESM.pdf]

Description of Additional Supplementary Files

File Name: Supplementary Data 1
Description: **Characteristics of the study participants included in the discovery and replication analyses**

File Name: Supplementary Data 2
Description: **List of 273 SNPs included in the study.** These are SNPs with P<5 x10-8 in Warrington et al 2019 and available in the NICHD Fetal Growth Studies dataset. Signal number="b" for SNPs with 6.6×10^{-9} <P< $5x10^{-8}$

File Name: Supplementary Data 3
Description: **Significant cis-eQTL associations between birthweight-associated single nucleotide polymorphisms and gene expression in placenta. Association.** P values are based on a twosided linear regression and have been corrected for multiple testing at 5% FDR.

File Name: Supplementary Data 4
Description: **Significant cis-mQTL associations between birthweight-associated single nucleotide polymorphisms and DNA methylation in placenta.** P values are based on a two-sided linear regression and have been corrected for multiple testing at 5% FDR.

File Name: Supplementary Data 5
Description: **Causal Inference Test (CIT) results for 197 eQTL-mQTL triplets**. TSS stands for transcription start site. Presented nominal P values are two-sided test.

File Name: Supplementary Data 6
Description: **MR Steiger test results for 197 eQTL-mQTL triplets.** TSS stands for transcription start site. P values are two-sided and have been corrected for multiple testing at 5% FDR.

File Name: Supplementary Data 7
Description: **Summary description of gene expression, tissue specificity, predicted location of protein expression, and molecular and protein function**

File Name: Supplementary Data 8
Description: **Replication moloc results in blood eQTL and mQTL, results with suggestive evidence of colocalization (PPA>=0.5).** TSS stands for transcription start site

File Name: Supplementary Data 9
Description: **Results of colocalization test in GTEx tissues.** PPA-H4=posterior probability of association. Hypothesis 4 (coloc2 results from LocusFocus, https://locusfocus.research.sickkids.ca/)

File Name: Supplementary Data 10
Description: **Independent replication of eQTLs and mQTLs in the RICHS cohort.** TSS stands for transcription start site. In bold are associations with P-value < 0.05. *rs77553582 (chr7:35299657) was missing in RICHS, and was replaced by rs7790713 (chr7:35299841) which is in perfect LD (r2=1 in Europeans). Empty cells denote that either the respective CpG site, SNP or gene was not present in RICHS after quality control. Presented nominal P values are based on a two-sided linear regression test.

File Name: Supplementary Data 11

Description: **Association of placental expression of genes identified in multi-trait colocalization with birthweight and placenta weight.** Model is based on linear regression adjusting for fetal sex and gestational age at delivery in RICHS (n=200) and NICHD Fetal Growth Studies (n=75). For birthweight, random-effects meta-analysis was performed on genes available in both datasets. For placenta weight, only results from NICHD Fetal Growth Studies were presented because placenta weight is absent in RICHS. In bold are directionally consistent associations in both datasets, and in red are associations with P-value< 0.05. Presented nominal P values are based on a two-sided linear regression test.

File Name: Supplementary Data 12
Description: **Significantly enriched (FDR< 0.05) mQTL target CpGs (n=778 CpG sites) for DHS, 15-state chromatin marks, and five H3 histone marks.** P-values are based on a one-sided hypergeometric test and have been corrected for multiple testing at 5% FDR.

File Name: Supplementary Data 13
Description: **Enrichment of mQTL target CpGs (n=778 CpG sites) for CpG island features.** In bold are associations with FDR P-value <0.05. P values are based on a one-sided hypergeometric test and have been corrected for multiple testing at 5% FDR.

File Name: Supplementary Data 14
Description: **mQTL-related CpGs reported to be associated with traits as reported by the catalog of epigenome-wide association studies.** P values are presented as reported by each study deposited in the catalog.

File Name: Supplementary Data 15
Description: **Enriched Cannonical Pathways from Ingenuity Pathway Analysis for genes annotating the mQTL SNPs and their target DNAm sites (a set of 402 genes) and genes annotating the eQTL SNPs and their target eGenes (a set of 50 genes).** P-values are based on a one-sided Fisher exact test and have been corrected for multiple testing at 5% FDR.

File Name: Supplementary Data 16
Description: **Enrichment of input genes in hallmark gene sets defined by the Molecular Signatures Database (MsigDB v7.2) via FUMA.** Shown here are significantly enriched gene sets (based on a one-sided over-representation test corrected for multiple testing at FDR P< 0.05)
